# Development of a TaqMan Real-Time PCR for Early and Accurate Detection of Anthracnose Pathogen *Colletotrichum siamense* in *Pachira glabra*

**DOI:** 10.3390/plants13081149

**Published:** 2024-04-20

**Authors:** Jiaqi Gu, Haihua Wang, Xiaoyan Huang, Limei Liao, Huan Xie, Xixu Peng

**Affiliations:** 1 School of Life and Health Sciences, Hunan University of Science and Technology, Xiangtan 411201, China; 21020901002@mail.hnust.edu.cn (J.G.); 22010901021@mail.hnust.edu.cn (X.H.); 22010901022@mail.hnust.edu.cn (L.L.); 22010901014@mail.hnust.edu.cn (H.X.); 2 Key Laboratory of Genetic Improvement and Multiple Utilization of Economic Crops in Hunan Province, Xiangtan 411201, China; 3 Key Laboratory of Integrated Management of the Pests and Diseases on Horticultural Crops in Hunan Province, Xiangtan 411201, China; 4 Key Laboratory of Ecological Remediation and Safe Utilization of Heavy Metal-Polluted Soils in Hunan Province, Xiangtan 411201, China

**Keywords:** *Pachira glabra*, anthracnose, *Colletotrichum siamense*, TaqMan real-time PCR, pathogen detection

## Abstract

Anthracnose, caused by *Colletotrichum siamense*, is a destructive disease of *Pachira glabra* in southern China. Early and proper monitoring and quantification of *C. siamense* is of importance for disease control. A calmodulin (*CAL*) gene-based TaqMan real-time PCR assay was developed for efficient detection and quantification of *C. siamense*, which reliably detected as low as 5 pg of genomic DNA and 12.8 fg (5800 copies) of target DNA. This method could specifically recognize all tested *C. siamense* isolates, while no amplification was observed in other closely related *Colletotrichum* species. The assay could still detect *C. siamense* in plant mixes, of which only 0.01% of the tissue was infected. A dynamic change in the amount of *C. siamense* population was observed during infection, suggesting that this real-time PCR assay can be used to monitor the fungal growth progression in the whole disease process. Moreover, the method enabled the detection of *C. siamense* in naturally infected and symptomless leaves of *P. glabra* trees in fields. Taken together, this specific TaqMan real-time PCR provides a rapid and accurate method for detection and quantification of *C. siamense* colonization in *P. glabra*, and will be useful for prediction of the disease to reduce the epidemic risk.

## 1. Introduction

*Pachira glabra Pasq.*, well known as the money tree or the lucky tree, is a medium-sized perennial evergreen tree belonging to the Malvaceae family. Being a native species of the Brazilian Atlantic Forest, it was introduced to China for landscaping use due to its ornamental value. The tree grows fast, and has a light and soft wood, which can be applied to manufacture light objects such as boxes, toys, and stationery stuff [1]. Its mature seeds, young leaves, and flowers are edible [2]. Moreover, the leaves of *Pachira glabra*, known to contain phenolic acids, quercetin flavonols, and glucoside derivatives, have been a traditional source for local people for some time, and used as a traditional remedy for stomach problems, ulcers, and headache [3]. With the continuous increase of plantation area in southern China, *P. glabra* is frequently decimated by fungal diseases, causing enormous economic losses. In our previous studies, white leaf spot and anthracnose, caused by *Hypomontagnella monticulosa* and *Colletotrichum siamense*, respectively, were frequently observed in *P. glabra* trees [4,5]. Recently, leaf brown spot, caused by *Diaporthe phoenicicola*, has been observed on *P. glabra* by our lab [6]. An anthracnose was observed with the incidence of up to 30% on *P. glabra* leaves. *P. glabra* anthracnose develops into sunken, necrotic areas, eventually leading to early leaf death and abscission, thus seriously affecting the quality of *P. glabra*. The disease exhibits leaf symptoms similar to those observed for leaf spot caused by *Diaporthe pachirae* [1], thus visual disease diagnosis may be unreliable.

The causal agent of *P. glabra* anthracnose, *Colletotrichum siamense*, belongs to the *C. gloeosporioides* species complex (CGSC), which comprises about 40 or more closely related species [7,8,9,10]. Species within the CGSC represent major pre- and post-harvest fungal pathogens causing anthracnose in diverse economically important crops of tropical and subtropical origin [7,11,12]. Traditionally, species within the CGSC have been differentiated based on morphological and cultural characters. However, species delimitation within the CGSC is challenging due to a lack of distinctive morphological features available for identification [11,13]. On the other hand, morphological characters vary with growth conditions, and are often not reliable enough to be used for species differentiation [14]. Thus, species determination of the CGSC mainly relies on molecular systematic analysis. Several gene markers are used for the molecular identification, including the nuclear rDNA internal transcribed spacers (*ITS*), actin (*ACT*), chitin synthase (*CHS-1*), β-tubulin (*TUB2*), calmodulin (*CAL*), glyceraldehydes-3-phosphate dehydrogenase (*GAPDH*), translation elongation factor 1-α (*TEF1*), glutamine synthetase (*GS*), DNA lyase (*APN2*), and the intergenic region between the *APN2* and *MAT1-2-1* genes (*APN2/MAT-IGS*, also referred as *ApMat*) [14].

CGSC species are well known to exhibit hemibiotrophic lifestyles, and can persist on host tissues without causing visible symptoms [15]. Discernible lesions often occur late in the host–pathogen interaction, making it difficult to quantify the fungal growth at the early stage of infection. The early and proper monitoring and quantification of pathogens is of importance for disease control. Therefore, rapid and reliable molecular diagnostic assays are essential for detecting phytopathogenic CGSC species in suspected plant material at early growth stages so that effective disease control strategies can be implemented to prevent the dissemination of diseases. 

Generally, traditional diagnosis of plant pathogens depends on visual observation, microscopy, mycological analysis, and biological diagnostics [16]. These methods are laborious, time-consuming, and require substantial expertise. Real-time PCR offers a fast, accurate, and culture-independent tool, and is widely used for the detection of plant pathogens [17,18,19,20]. Compared with other real-time PCR assays, the TaqMan real-time PCR is more specific, sensitive, and accurate. Owing to these prominent advantages, the TaqMan real-time PCR has been used to detect a variety of phytopathogenic CGSC species, including *C. theobromicola* on boxwood (*Buxus* spp. L.) [17], *C. lindemuthianum* on navy bean [21], *C. lagenarium* on Arabica coffee [22], *C. karstii* [23], *C. orbiculare* on watermelon [24], and anthracnose causal agents *Colletotrichum* spp. on Chinese fir [25]. Recently, Du et al. [18] have developed an effective SYBR Green real-time PCR method targeting the *ITS* sequence for quantitative detection of *C. siamense* in rubber trees. However, phylogenetic analyses using the *ITS* marker reveal a low resolution for species discrimination and diagnosis within the CGSC [14], suggesting attention should be paid to its application on the molecular diagnosis of phytopathogens within this species complex. 

The present study aimed to develop a TaqMan real-time PCR assay for rapid detection of *C. siamense*, the causal agent of anthracnose on *P. glabra*. The specificity, sensitivity, and validity of the real-time PCR using a TaqMan probe targeting the *CAL* gene were evaluated using fungal culture, artificially inoculated *P. glabra* leaves, and naturally infected ones, demonstrating the applicability of this novel molecular diagnostic assay in field testing and disease management.

## 2. Results

### 2.1. Specificity of Primer Set and TaqMan Probe

**Figure 1:**
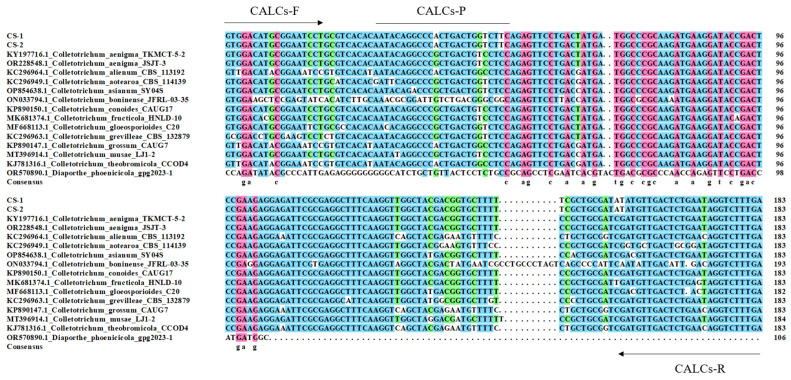
Alignment of calmodulin (*CAL*) sequences of *Colletotrichum siamense*, other closely related *Colletotrichum* species, and *Diaporthe phoenicicola*, highlighting the position of the primers CALCs-F/CALCs-R (arrows) and probe CALCs-P (line) designed for detecting *C. siamense* by TaqMan real-time PCR. Gaps are marked as dashes in sequences, and rose red, blue, and green boxes represent 100%, ≥75%, and ≥50% of nucleotide identity among the aligned sequences, respectively.

The retrieved or amplified CAL sequences of twenty-two fungi isolates (Table 1), including eight *C. siamense* isolates, thirteen closely related nontarget *Colletotrichum* species, and one nontarget *D. phoenicicola*, the casual agent of leaf brown spot on *P. glabra* [6], were used for multiple sequence alignments, and the *CAL* regions that are conserved for *C. siamense* isolates but different from the nontarget fungi were used to design specific primers and the TaqMan probe. All the selected *CAL* regions of the eight *C. siamense* isolates are the same, although several nucleotide polymorphisms do exist out of the aligned region. Thus, representative *C. siamense* isolates, i.e., CS-1 and CS-2, were included in the multiple sequence alignments. The results showed 7 to 24 nucleotide differences when compared with the closely related *Colletotrichum* species (Figure 1). Thus, based on the nucleotide differences of the *CAL* regions, the primer set CALCs-F/CALCs-R (5′-GTGGACATGCGGAATCCT-3′ and 5′-TCAAAGACCTATTCAGAGTCAACATAT-3′, respectively) and TaqMan probe CALCs-P (5′-AATACAGGCCCACTGACTGGTCTTC-3′) were designed for molecular determination of these species. BLASTn (https://blast.ncbi.nlm.nih.gov/Blast.cgi?PROGRAM=blastn&PAGE_TYPE=BlastSearch&LINK_LOC=blasthome, accessed on 3 July 2022) analysis of the designed primers and probe against the NCBI database showed that the primers CALCs-F/CALCs-R were specific to *C. siamense* and there were not any identical or highly similar sequences in any other sequenced organisms. 

**Figure 2:**
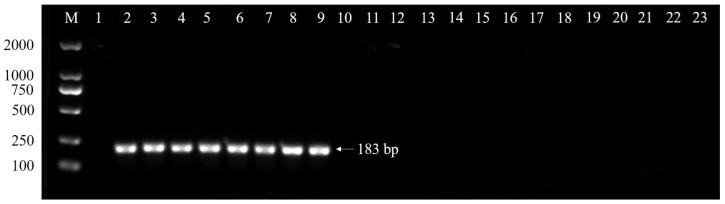
Conventional PCR amplification (amplicon size 183 bp) of *C. siamense* DNA (lane [L] 2 to L9) isolated from 7-day-old cultures and nonamplification of other closely related *Colletotrichum* species (L10 to L22) and *D. phoenicicola* (L23) using species-specific primers CALCs-F/CALCs-R. M = marker (100 to 2000 bp); L1 = nontemplate control; L2 = CS-1; L3 = CS-2; L4 = CS-3; L5 = CS-4; L6 = CS-5; L7 = CS-6; L8 = CS-7; L9 = CS-8; L10 = TKMCT-5-2 (*C. aenigma*); L11 = JSJT-3 (*C. aenigma*); L12 = CBS 113192 (*C. alienum*); L13 = CBS 114139 (*C. aotearoa*); L14 = SY04S (*C. asianum*); L15 = JFRL-03-35 (*C. boninese*); L16 = CAUG17 (*C. conoides*); L17 = HNLD-10 (*C. fructicola*); and L18 = C20 (*C. gloeosporioides*); L19 = CBS 132879 (*C. grevilleae*); L20 = CAUG7 (*C. grossum*); L21 = LJ1-2 (*C. musae*); L22 = CCOD4 (*C. theobromicola*); and L23 = gpg2023-1 (*Diaporthe phoenicicola*).

**Figure 3:**
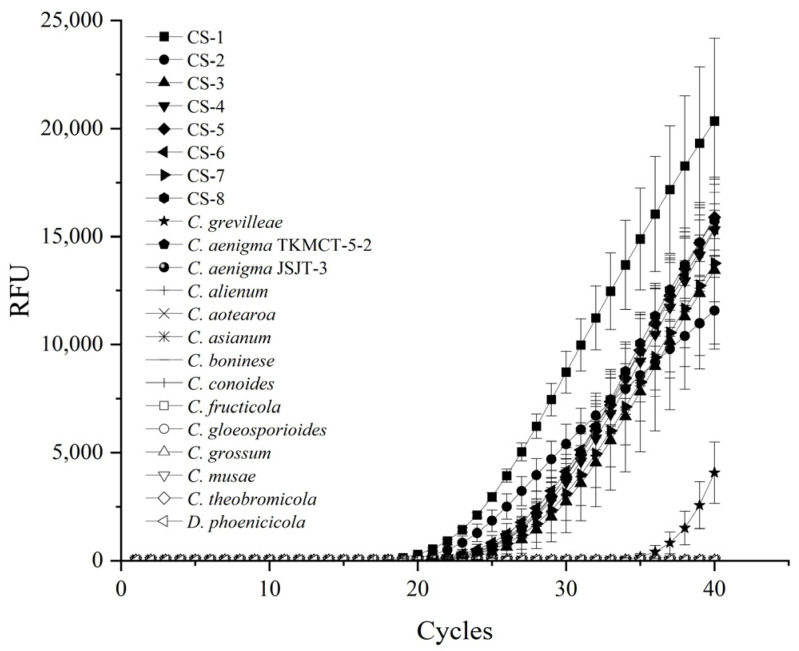
Amplification curves of *Colletotrichum siamense* DNA from eight isolates (1–8) using species-specific primers CALCs-F/CALCs-R and TaqMan CALCs-P probe. No amplification was observed from DNA samples isolated from nontarget fungi *C. grevilleae*, *C. aenigma*, *C. alienum*, *C. aotearoa*, *C. asianum*, *C. boninese*, *C. conoides*, *C. fructicola*, *C. gloeosporioide*, *C. grossum*, *C. musae*, *C. theobromicola* and *Diaporthe phoenicicola* (9–22). The data represent mean values ± SE calculated from three replicates.

The specificity of the primers CALCs-F and CALCs-R was first analyzed by a conventional PCR. The primers set amplified a 183-bp PCR product, confirmed through sequencing, with DNA extracted from *C. siamense* cultures. No amplification was observed for other closely related *Colletotrichum* species, including *C. aenigma*, *C. alienum*, *C. aotearoa*, *C. asianum*, *C. boninese*, *C. conoides*, *C. fructicola*, *C. gloeosporioide*, *C. grevilleae*, *C. grossum*, *C. musae*, *C. theobromicola*, *Diaporthe phoenicicola* (the causal agent of *P. glabra* leaf brown spot), and the nontemplate control (Figure 2). Then, the specificity of the primer set and TaqMan probe was checked using a TaqMan real-time PCR. The results showed that positive signals were obtained only from the target fungi species, but not from the nontarget ones (Table 1, Figure 3), indicating the primer set CALCs-F/CALCs-R and TaqMan probe CALCs-P were specific to *C. siamense*. The Ct values of the eight *C. siamense* isolates were 22–28 cycles, while those of the thirteen nontarget *Colletotrichum* species were more than 39 cycles (Table 1). The Ct values of the target and nontarget fungi were well separated into two different groups. These observations confirmed the specificity of the primer set and TaqMan probe for target species.

### 2.2. Sensitivity and Stability of the TaqMan Real-Time PCR Assay

**Figure 4:**
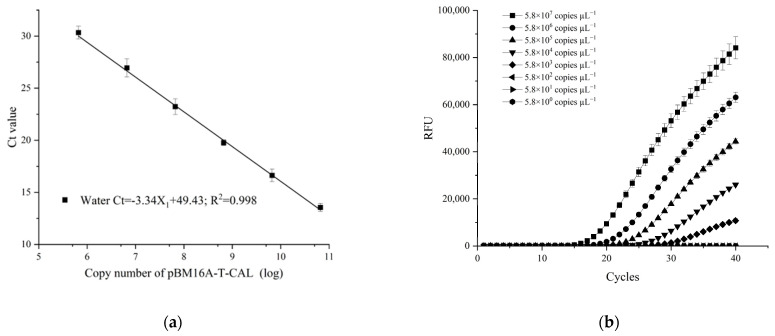

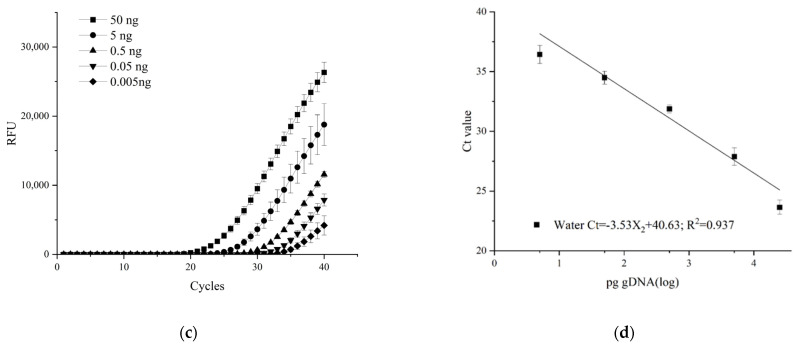
The TaqMan real-time PCR assays on *Colletotrichum siamense* CS-1 pDNA (pBM16A-T-CAL) (ranging from 5.8 × 10^0^ to 5.8 × 10^7^ copies μL^−1^) and gDNA (ranging from 0.005 to 50 ng μL^−1^) diluted with sterile water. (**a**) The standard curve for *C. siamense* CS-1 pDNA. (**b**) Amplification curves for *C. siamense* CS-1 pDNA. (**c**) Amplification curves for *C. siamense* CS-1 gDNA. (**d**) The standard curve for *C. siamense* CS-1 gDNA. The data represent mean values ± SE calculated from three replicates.

A standard curve was plotted by tenfold serial dilution of cloned target DNA (pDNA) ranging from 10^0^ to 10^7^ copies/µL (Figure 4a). The standard curve showed that there was a strong linear correlation between the logarithm of the pDNA concentrations and Ct values, with high correlation coefficient (R^2^ = 0.998) and high amplification efficiency (E = 99.3%). The amplification curves of pDNA showed a reliable detection limit down to 1, 000 copies (Figure 4b), the sensitivity of which was 100 times higher than that of a conventional PCR (Figure S1). 

Five tenfold serially diluted gDNA of *C. siamense* with ddH_2_O, ranging from 20 ng to 5 pg, were also evaluated using this target gene. The regression curve generated with a slope value of −3.53 (E = 91.9%, R^2^ = 0.937). The lowest detection level with fungal gDNA was determined to be 5 pg (Figure 4c,d).

**Figure 5:**
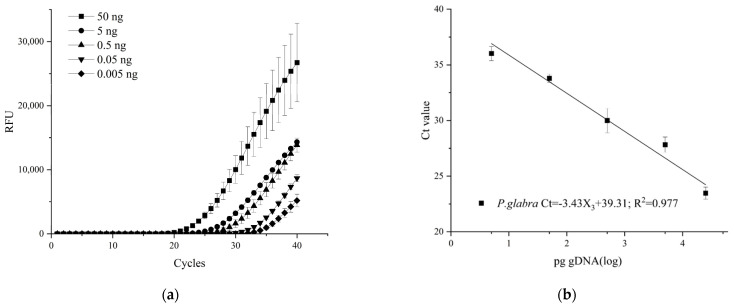
The TaqMan real-time PCR assays on *Colletotrichum siamense* CS-1 gDNA (ranging from 0.005 to 50 ng) diluted with the DNA extract of *Pachira glabra* leaves. (**a**) Amplification curves. (**b**) The standard curve. The data represent mean values ± SE calculated from three replicates.

To evaluate the potential effect of leaf extract on quantitative results, 1 μL of *C. siamense* CS-1 gDNA (5 ng/μL) was serially diluted with *P. glabra* leaf extract (0, 0.5, 1, 1.5, 2, 2.5 μL). Statistical analysis showed that there were no statistically significant amplification results regardless of the presence or absence of host leaf extract in the *C. siamense* isolate CS-1 gDNA (*p* = 0.785 > 0.05) (Table 2). Therefore, the TaqMan real-time PCR was not significantly affected by plant background using primer set CALCs-F/CALCs-R and TaqMan probe CALCs-P. A standard curve was generated by serial dilution of 50 ng gDNA of fungal mycelia with DNA of healthy leaves, resulting in a slope of −3.43 and R^2^ value of 0.977 with an E value of 95.7% (Figure 5). The lowest detection level with infected plant DNA tissue was determined to be 5 pg/μL.

### 2.3. Detection in Artificially Inoculated P. glabra

**Figure 6:**
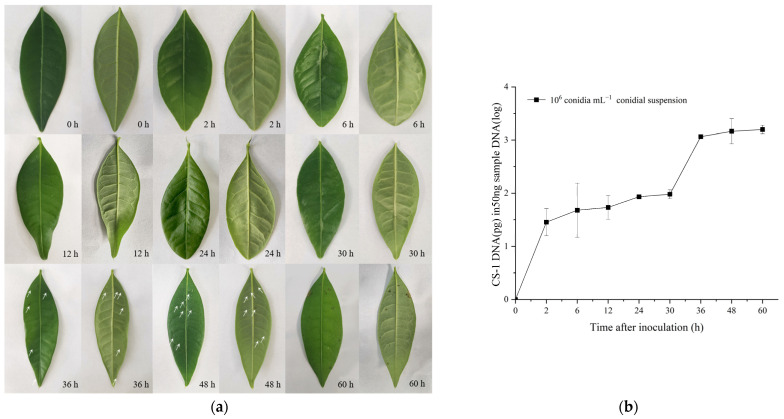
*Colletotrichum siamense* time-course infection on *Pachira glabra* leaves during 0 to 60 h post inoculation with 10^6^ conidia/mL. (**a**) Photographs of leaves infected. The white arrows indicate disease spots at the onset times. (**b**) *C. siamense* DNA quantification. The data represent mean values ± SE calculated from three replicates as quantified by TaqMan real-time PCR.

The disease progression of *C. siamense* in *P. glabra* leaves after artificial inoculation was monitored with visual observations and real-time PCR analyses. During the first 24 h post inoculation (hpi), *P. glabra* leaves remained symptomless (Figure 6a). However, the amount of *C. siamense* DNA steadily increased by 28.5 to 85.7 pg per 50 ng DNA of leaves over this period (Figure 6b). These findings suggest that the real-time PCR method allows the monitoring of growth progression during the latent phase. During the symptomatic stage of the infection, the amount of *C. siamense* DNA largely increased over time in the inoculated leaves. Small black spots first occurred sporadically at 36 hpi, when the *C. siamense* DNA was 1159.2 pg per 50 ng DNA of *P. glabra* leaves. By 60 h, leaf spots became denser and larger, and several began to coalesce. The amount of *C. siamense* DNA reached 1578.52 pg per 50 ng of total DNA at this time. These results showed that the TaqMan real-time PCR method could monitor the growth progression of *C. siamense* in the whole process of disease.

### 2.4. Detection in Naturally Infected P. glabra

*C. siamense* was detected for naturally infected leaves in fields by real-time PCR. For the 25 asymptomatic but suspected to be infected leaves, fluorescent signals were detected in seven leaves with an average DNA content of 85.11 pg per 50 ng DNA sample, suggesting that anthracnose symptoms would probably be developed in these seven asymptomatic leaves. No amplification was observed for DNA extracted from the other 18 asymptomatic leaves (Table 3). The seven asymptomatic leaves were also tested by conventional PCR. Only two leaves exhibited positive amplifications, indicating the conventional PCR method was less sensitive than the real-time PCR. Fluorescent signals occurred with DNA extracted from naturally infected leaves. There was a positive correlation between disease severity and amount of fungal pathogen DNA. *C. siamense* was detectable in these samples by tissue isolation.

## 3. Discussion

Species within the CGSC cause destructive anthracnose diseases on a wide variety of economically valuable crops [7,11,12]. There are obstacles to differentiate and detect CGSC species in diseased plant materials, including limited morphological differentiation among species, variation in pathogenicity and cultural morphology, and common latency or quiescence in host cells [15,26]. Thus, the molecular method is expected to supply an ideal solution for disease diagnosis. It is essential to accurately and rapidly identify CGSC species that cause anthracnose disease so as to develop effective control strategies. In this study, the TaqMan probe-based real-time PCR assay using *CAL* marker provides a specific, sensitive, and effective method for detecting *C. siamense*, the causal agent of *P. glabra* anthracnose; thus, it is suitable for application in early prediction of *P. glabra* anthracnose to reduce the epidemic risk. 

Successful detection of pathogens by PCR-based methods needs primers to satisfy the criteria, including uniqueness of the amplification sequence to the target pathogen of interest and conservation across populations of the target pathogen [27]. Several molecular protocols based on real-time PCR amplification have been previously developed for detection and quantification of *Colletotrichum* species, targeting different molecular markers such as *ITS* [18,21], *GAPDH* [22], *CAL* [17], *ACT* [20], cutinase (*Cuti*) [19], and *ApMat* [25]. In this study, real-time PCR primers and a probe specific to *C. siamense* were designed based on the sequence differences in the *CAL* gene region. The TaqMan real-time PCR method accurately identified and quantified *C. siamense* using DNA from pure cultures, a DNA mixture of the target fungus and plant tissues, and DNA from the infected *P. glabra* leaves. This assay is species specific, as indicated by lack of amplification of nontarget species (Figure 2 and Figure 3). Systematic analyses of phylogenetic informativeness profiling and Bayesian concordance using various molecular markers reveal that *APN2/MAT-IGS*, *APN2*, the intergenic spacer between *GAPDH,* and a hypothetical protein (*GAP2-IGS*), and the longest *TUB2* are the most suitable gene markers for reliably discriminating species within the CGSC, while *CAL*, along with *GAPDH* and *GS*, has intermediate informativeness values [14,28]. However, the *CAL* gene does contain unique sequence motifs that can be used to successfully differentiate *C. siamense* from other closely related species in this study. A similar study was conducted by Kaur et al., who developed a diagnostic TaqMan real-time PCR assay for accurately detecting and quantifying *C. theobromicola* in boxwood using the *CAL* marker [17]. These assays suggest that molecular targets with relatively intermediate resolution for large genera like *Colletotrichum* could still be used for identification and qualification of a given species within the genera, if appropriate primers and probes are designed with special care.

The effectiveness of TaqMan real-time PCR assays relies on not only high specificity, but also on high sensitivity of primers and a probe that could specifically amplify the target sequences. In the present study, an optimized protocol of a TaqMan real-time PCR assay was proposed and used to diagnose and quantify *C. siamense*, the causal agent of *P. glabra* anthracnose, at very low concentrations of fungal DNA. The assay could reliably detect as low as 12.8 fg/µL (5.8 × 103 copies/µL) of target DNA, 5.0 pg/µL of gDNA, and *C. siamense* in plant mixes, of which 0.01% of the total tissue DNA was pathogen DNA. This detection threshold is comparable to that of the *CAL*-based real-time TaqMan PCR technology for detection of *C. theobromicola* in boxwood [17], and slightly lower than that of the *CAL*-targeted loop-mediated isothermal amplification (LAMP) for detection of *C. siamense* in infected tea plants [29]. However, LAMP could not provide quantitative data of target DNA, so it is not suitable for monitoring the dynamics of the pathogen [30]. The detection limit of the present assay targeting the *CAL* gene was higher than that of the assay targeting the *ApMat* gene in Chinese fir (1 ng/µL of gDNA) and lower than the assay targeting the multi-copy *ITS* in rubber trees (100 fg/µL of gDNA) reported previously [18,25]. This might be attributed to the different copies of these gene markers in the fungal genomes. The *ITS* region has been suggested to represent the universal barcode for fungi [31] but with several concerns [32]. However, the utility of this region is limited for systematic identification and delimitation in *Colletotrichum* [14]. Bhunjun et al. evaluated the phylogenetic significance of five molecular markers for species delineation within each species complex of *Colletotrichum*, and found that the *ITS* region was the least parsimony informative locus [33]. We attempted to design the primers and probe of TaqMan real-time PCR based on the *ITS* to differentiate and quantify *C. siamense* from the other closely related *Colletotrichum* species listed in Table 1, and no satisfactory amplification signals were obtained. The efficiency of the present assay using both fungal DNA in water and the same DNA spiked with DNA from *P. glabra* leaves was within the acceptable limits from 91.9% to 99.3% (−3.53 > slope > −3.34) (Figure 4d and Figure 5b). Similar efficiencies were displayed in the real-time assays for detection of *C. theobromicola* in boxwood and *C. siamense* in rubber trees [17,18]. Accordingly, the sensitivity and PCR efficiency of the present method are sufficient for accurate detection and quantification of *C. siamense* in *P. glabra* trees.

Some compounds introduced from host plants during DNA extraction, such as polysaccharides and phenolic substances, are potential PCR inhibitors [31]. A prerequisite for in planta DNA quantification is to avoid the potential interference of plant tissues in detection and quantification of pathogens. As suggested by Du et al. [18], DNA extracts of plant material were diluted to 50 ng/µL in this study. The results showed the dilution level could preclude any bias due to PCR inhibitors originating from plant tissues. Accordingly, the presence of *P. glabra* leaf samples did not affect the efficiency, sensitivity, and reproducibility of the real-time PCR assays. 

*C. siamense* is a hemibiotrophic fungus that can remain in a latent state in host tissues [15]. At the latent stage, no visible difference can be observed between healthy and infected tissues. Therefore, early and accurate detection is critical for controlling this fungus known to cause delayed discernable symptoms in host plants. In the present study, the presence of *C. siamense* was successfully detected by using the real-time PCR assay in artificially inoculated leaves at 2 hpi, while the first visible lesion usually occurred at 36 hpi (Figure 6a). The results indicate that this assay can be used to detect *C. siamense* in the latent phase in host leaves. Moreover, the two-stage induction of *C. siamense* DNA was observed during the infection process (Figure 6b), which was consistent with visual observation, confirming that this method is useful for monitoring the dynamics of *C. siamense* in *P. glabra* trees. 

Finally, the effectiveness and potential application of the real-time assay were validated for *C. siamense* detection in naturally infected *P. glabra* leaves collected from fields. The infection was also confirmed by the tissue isolation method. The amount of *C. siamense* DNA detected by the real-time assay correlated positively with the disease severity (Table 3). Therefore, the TaqMan PCR technique presented here is an efficient detection strategy for predicting and monitoring the epidemics of *C. siamense* in fields.

## 4. Materials and Methods

### 4.1. Fungal Isolates

In total, eight isolates of *C. siamense* from *P. glabra* and fourteen isolates of nontarget species from various hosts and geographical regions were used in this study (Table 1). The *C. siamense* isolates used in this study were obtained from the infected *P. glabra* plants collected from three provinces in China, including Hunan [5], Guangxi, and Guangdong. The nontarget isolates included 13 other closely associated *Colletotrichum* species and one isolate of *Diaporthe phoenicicola* gpg2023-1 [6], which causes leaf brown spots on *P. glabra* as mentioned above. The fungal isolates were revived on potato dextrose agar (PDA) plates at 28 °C under dark conditions for 5 to 7 days. Then, mycelial plugs taken from the edge of fungal colonies were transferred to new PDA plates under the same conditions.

### 4.2. Plant Growth

About one-year-old *P. glabra* plants purchased from a local nursery were planted in peaty soil, and cultivated in a greenhouse at 25 °C under natural sunlight. For leaf inoculation experiments, healthy individuals were moved into a growth chamber at 25 °C for another 14 days before inoculation. The parameters were set as follows: 12-h photoperiod per day, photo flux density 125 μmol m^−2^ s^−1^, relative humidity 80 to 85%.

### 4.3. DNA Extraction

Genomic DNA (gDNA) of 5-to-7-day-old fungal mycelia and 0.5 g of plant material were extracted according to the instructions of the Biospin Fungal Genomic DNA Extraction Kit (BioFlux, Hangzhou, China). DNA samples were stored at −20℃ for use. The quality and quantity of DNA samples were determined by ultra-micro spectrophotometer (NanoPHotometer-NP80; Implen GmbH, Munich, Germany) at the ratio of absorption at 260 and 280 nm.

### 4.4. Cloning of the Target Sequence

The partial *CAL* sequence of *C. siamense* isolate CS-1 was amplified using primer set CALCs-F (5′-GTGGACATGCGGAATCCT-3′) and CALCs-R (5′-TCAAAGACCTATTCAGAGTCAACATAT-3′). The PCR amplification was performed in a 20 µL-reaction volume containing 10 µL of 2 × Taq Mix (Vazyme, Nanjing, China), 0.4 µL of each forward and reverse primer (10 µmol/L), 1.0 µL of gDNA (100 ng/µL), and 8.2 µL of sterile double-distilled water (ddH_2_O). The PCR reaction parameters were set as follows: initial denaturation at 95 °C for 3 min followed by 35 cycles of 30 s at 95 °C, 30 s at 57 °C, 20 s at 72 °C, and a final extension at 72 °C for 5 min. The PCR products were visualized on a 2% agarose gel run at 110 V for 30 to 40 min, and their identities were confirmed by DNA sequencing (Jieteng Biological Co., Kunming, China). 

The expected fragments were gel-purified with a Gel Extraction Kit (BioFlux, Hangzhou, China), and ligated to pBM16A-T vector with a pBM16A Toposmart Cloning Kit (Biomed, Beijing, China). The resulting pBM16A-T-CAL plasmids were transferred into *Escherichia coli* DH5α, extracted using a Biospin Plasmid DNA Mini Extraction Kit (BioFlux, Hangzhou, China), and confirmed by sequencing (Jieteng Biological Co., Kunming, China). The plasmid DNA (pDNA) concentration was measured with an ultra-micro spectrophotometer and converted to copy number according to the formula:

Number of copies = (amount × 6.022 × 10^23^)/(length × 10^9^ × 650). (1)

### 4.5. Real-Time Primers and TaqMan Probe Design

Known sequences of the *CAL* gene region were retrieved from the GenBank database (https://www.ncbi.nlm.nih.gov/). The *CAL* sequences of *C. siamense* from the isolates CS-2 to CS-8 and *D. phoenicicola* gpg2023-1 were amplified as described above. These sequences were aligned and compared using MEGA11 with default parameters, and the regions of nucleotide differences in the *CAL* sequences between target species and nontarget ones were used to design the real-time PCR primers and TaqMan probes using Primer premier 5.0 (PREMIER Biosoft International, San Francisco, CA, USA). The probe was labeled at the 5’ end with 6-carboxyfluorescein (FAM) as a reporter dye, and modified at the 3’ end with the quencher dye tetramethylcarboxyrhodamine (TAMRA). The primers and probe were synthesized by Jieteng Biological Co. (Kunming, China). 

The specificity of primer set was evaluated by a conventional PCR as described above, and further verified by amplification curve analysis of a real-time PCR as described below. The specificity of the TaqMan probe was assessed by a real-time PCR. Both the conventional PCR and real-time PCR were conducted with 50 ng gDNA of the target and nontarget species as templates.

### 4.6. TaqMan Real-Time PCR Assay

The real-time PCR assays were performed in a total volume of 20 µL containing 10 µL of 2× TaqMan Mix (BioFlux, Hangzhou, China), 0.4 µL of each forward and reverse primer, and 1.8 µL of the TaqMan probe, 1 µL of DNA templates (50 ng/µL), 0.2 µL of Taq polymerase (5 U/µL) (BioFlux, Hangzhou, China), and 6.2 µL of sterile ddH_2_O. The CFX96 Real-Time System Thermocycler (Bio-Rad Laboratories, Hercules, CA, USA) was used for amplification and fluorescence measurement. The thermocycling conditions were as follows: initial denaturation at 95 °C for 2 min, followed by 40 cycles of denaturation at 95 °C for 10 s, then annealing and extension at 63.9 °C for 30 s. Signal threshold levels were set automatically by the system. Triplicate reactions were performed in each assay and each assay was repeated at least twice.

### 4.7. Sensitivity and Stability of the TaqMan Real-Time PCR Assay

To evaluate the sensitivity of the TaqMan real-time PCR assay, standard curves were generated by amplifying 10-fold serial dilutions of pDNA (pBM16A-T-CAL) ranging from 10^0^ to 10^7^ copies/µL. Sterilized ddH_2_O instead of template DNA was served as negative control. Triplicate reactions were performed in each assay, and each assay was repeated at least twice. Each run contained a negative control using sterile ddH_2_O instead of DNA templates. The samples with a stable Ct value and amplification curve ratio were selected to establish the linear regression curves between the logarithm of the template concentration and the Ct values. The PCR amplification efficiency (E) was calculated by the formula [34]:

E = (10^1/-slope^ − 1) × 100. (2)

To assess whether leaf extracts of *P. glabra* could interfere with the detection of *C. siamense*, a series of leaf extracts (0, 0.5, 1, 1.5, 2, and 2.5 µL) were mixed with 1 µL of 5 ng/µL gDNA and added into a total real-time PCR reaction volume of 20 μL. Leaf extracts were prepared according to the method described by He et al. [25]. The leaf extracts and sterile ddH_2_O were included in each real-time PCR run as negative controls. In the positive control, gDNA of *C. siamense* isolate CS-1 culture without *P. glabra* leaf extracts was used as a template. Triplicate reactions were performed in each assay and each assay was repeated at least twice. Standard curves were also generated by amplifying 10-fold serial dilutions of DNA extracted from the *P. glabra* leaves infected by *C. siamense* isolate CS-1 (50 to 0.005 ng/µL).

### 4.8. Validity of the TaqMan Real-Time PCR on Artificially Inoculated Plants

Conidial suspension of *C. siamense* isolate CS-1 were prepared from PDA cultures. The conidia were counted by a blood cell counting chamber. Conidial suspension (containing 0.7% glucose and 0.05% Tween 20) was sprayed on the leaves of healthy *P. glabra* plants until runoff. The inoculated plants were maintained at a growth chamber under the growth conditions described above. The disease symptoms of plants were observed and recorded at 2, 6, 12, 24, 30, 36, 48, and 60 hpi. Three leaves were randomly picked from the inoculated plants during each time, surface-sterilized through sequential immersion in 0.5% sodium hypochlorite then 75% ethanol, and thoroughly washed with sterilized water three times. Then, the leaves were dried with filter paper and frozen quickly with liquid nitrogen for DNA extraction and real-time PCR assays.

### 4.9. Validity of the TaqMan Real-Time PCR on Naturally Infected Plants in Fields

*P. glabra* leaves were sampled as described by Du et al. [18] with minor modifications. Briefly, 25 leaves without lesions but suspected to be infected with *C. siamense*, 3 naturally infected leaves for each disease grade, and healthy leaves were collected, and placed in sealed bags. Leaf DNA was extracted as described above. DNA samples were diluted to 50 ng/µL with sterile ddH_2_O for real-time PCR detection using DNA of healthy leaves as blank controls. The amount of *C. siamense* gDNA was estimated by comparing Ct values to the gDNA standard curve. *C. siamense* in plants was detected using a tissue separation method as described previously [5] and identified based on cultural and morphological characteristics [11].

Disease grades were determined based on six symptom grades as follows: 0 refers to no lesion, 1 refers to lesion area occupied <5% leaf area, 2 refers to lesion area occupied ≥5% but <10% leaf area, 3 refers to lesion area occupied ≥10% but <20% leaf area, 4 refers to lesion area occupied ≥20% but <40% leaf area, and 5 refers to lesion area occupied ≥40% leaf area or death of leaves.

### 4.10. Statistical Analysis

Data represent mean values ± standard errors (SEs) calculated from three replicates. Analysis of variance (one-way ANOVA) and multiple comparisons of differences between assays (Duncan’s multiple range test, *p* < 0.05) were performed using SPSS for Windows version 24.0 (SPSS Inc., Chicago, IL, USA).

## 5. Conclusions

In the current work, an efficient TaqMan real-time PCR assay has been developed for specific and accurate detection of *C. siamense* on the host *P. glabra* tree by targeting the *CAL* gene. This assay enables a rapid and reliable diagnostic method to detect *P. glabra* anthracnose at early stages of the disease, thus is proper for application in early prediction of the disease to reduce the risk of epidemics. This method can be used in a high-throughput selection of anthracnose-resistant *P. glabra* cultivars during germplasm identification, and also valuable for official phytosanitary purposes. The qPCR assay is about to enter into a contract with a private sector user.

## Figures and Tables

**Table 1:** Strains and origins of *Colletotrichum siamense* and other fungi species, and specificity of the TaqMan real-time PCR assays targeting calmodulin gene.

Sample No.	Species	Strains	Host	Origin	Ct Value
1	*C. aenigma*	TKMCT-5-2	*Trichosanthes kirilowii* Maxim	China	>40
2		JSJT-3	*Carya illinoinensis*	Jiangsu, China	>40
3	*C. alienum*	CBS 113192	*Protea cynaroides*	South Africa	>40
4	*C. aotearoa*	CBS 114139	*Knightia* sp.	New Zealand	>40
5	*C. asianum*	SY04S	*Mangifera indica* L.	Guangxi/Guizhou/Hainan, China	>40
6	*C. boninese*	JFRL-03-35	*Aucuba japonica*	Guizhou, China	>40
7	*C. conoides*	CAUG17	*Capsicum annuum*	Jiangsu, China	>40
8	*C. fructicola*	HNLD-10	*Fragaria ananassa*	China	>40
9	*C. gloeosporioides*	C20	*Robinia pseudoacacia*	Shandong, China	>40
10	*C. grevilleae*	CBS 132879	*Grevillea* sp.	Italy	39.2
11	*C. grossum*	CAUG7	*Coprosma* sp.	Hainan, China	>40
12	*C. musae*	LJ1-2	*Musa* spp.	Guangxi, China	>40
13	*C. siamense*	CS-1	*Pachira glabra*	Hunan, China	22.65
14		CS-2	*Pachira glabra*	Hunan, China	23.97
15		CS-3	*Pachira glabra*	Hunan, China	27.74
16		CS-4	*Pachira glabra*	Hunan, China	26.56
17		CS-5	*Pachira glabra*	Hunan, China	26.42
18		CS-6	*Pachira glabra*	Hunan, China	26.15
19		CS-7	*Pachira glabra*	Guangdong, China	27.86
20		CS-8	*Pachira glabra*	Hainan, China	26.65
21	*C. theobromicola*	CCOD4	*Gossypium hirsutum* L.	Guizhou/Hubei/Henan, China	>40
22	*D. phoenicicola*	gpg2023-1	*Pachira glabra*	Hunan, China	>40

**Table 2:** The effect of *Pachira glabra* leaf extract on the Ct value of the TaqMan real-time PCR assays.

Templates	*Pachira glabra* Extract (μL)	Ct (Mean ± SD)
5 ng CS-1 gDNA	2.5	28.61 ± 0.23
	2.0	29.50 ± 0.81
	1.5	29.74 ± 1.06
	1.0	28.10 ± 0.32
	0.5	28.43 ± 0.42
	0	27.95 ± 0.16
	*p* = 0.785	

**Table 3:** Detection of *Colletotrichum siamense* in symptomless and infected leaves of *Pachira glabra* in the field using the TaqMan real-time PCR and conventional PCR.

Disease Grade	Number of Leaves	CS-1 DNA Amount [log (pg per 50 ng of Sample DNA)]	Detection with Conventional PCR ^1^	Detection with the Tissue Isolation Method ^1^
0	18	0	ND	ND
	2	1.93 ± 0.42	ND	D
	5		D	D
1	3	2.65 ± 0.14	D	D
2	3	3.24 ± 0.07	D	D
3	3	3.46 ± 0.10	D	D
4	3	3.95 ± 0.09	D	D
5	3	4.14 ± 0.12	D	D

^1^ D, detected; ND, not detected. The data represent mean values ± SE calculated from three replicates.

## Data Availability

The datasets used and/or analyzed during the current study are available from the corresponding authors on request.

## Supplementary Materials

The following supporting information can be downloaded at: https://www.mdpi.com/article/10.3390/plants13081149/s1, Figure S1: PCR amplification of *C. siamense* CS-1 pDNA (pBM16A-T-CAL) (M, molecular DNA marker (100 to 2000 bp); NTC, nontemplate control).

## References

[1] Milagres C.A., Belisário R., Silva M.A., Lisboa D.O., Pinho D.B., Furtado G.Q. (2018). A novel species of *Diaporthe* causing leaf spot in *Pachira glabra*. Trop. Plant Pathol..

[2] Lawal O.A., Ogunwande I.A., Salvador A.F., Sanni A.A., Opoku A.R. (2014). *Pachira glabra* Pasq. essential oil: Chemical constituents, antimicrobial and insecticidal activities. J. Oleo Sci..

[3] El-Din M.I.G., Youssef F.S., Said R.S., Ashour M.L., Eldahshan O.A., Singab A.N.B. (2021). Chemical constituents and gastro-protective potential of *Pachira glabra* leaves against ethanol-induced gastric ulcer in experimental rat model. Inflammopharmacology.

[4] Tian J.H., Peng X.X., Wu Q.T., Wen B.Y., Deng C.C., Wang H.H. (2023). First report of white leaf spot caused by *Hypomontagnella monticulosa* on *Pachira glabra* in China. Plant Dis..

[5] Wu Q.T., Xiao Z.Y., Tian J.H., Li S.Q., Peng X.X., Wang H.H. (2023). First report of anthracnose caused by *Colletotrichum siamense* on *Pachira glabra* in China. Plant Dis..

[6] Deng C.C., Wang H.H., Wen B.Y., Gu J.Q., Peng X.X., Zhang Z.F. First report of leaf brown spot caused by *Diaporthe phoenicicola* on *Pachira glabra* in China. Plant Dis..

[7] Jayawardena R.S., Hyde K.D., Damm U., Cai L., Liu M., Li X.H., Zhang W., Zhao W.S., Yan J.Y. (2016). Notes on currently accepted species of *Colletotrichum*. Mycosphere.

[8] Khodadadi F., González J., Martin P.L., Giroux E., Bilodeau G.J., Peter K.A., Doyle V.P., Aćimović S.G. (2020). Identification and characterization of *Colletotrichum* species causing apple bitter rot in New York and description of *C. noveboracense* sp. nov. Sci. Rep..

[9] Sharma G., Maymon M., Freeman S. (2017). Epidemiology, pathology and identification of *Colletotrichum* including a novel species associated with avocado (*Persea americana*) anthracnose in Israel. Sci. Rep..

[10] Weir B.S., Johnston P.R., Damm U. (2012). The *Colletotrichum gloeosporioides* species complex. Stud. Mycol..

[11] Cannon P.F., Damm U., Johnston P.R., Weir B.S. (2012). *Colletotrichum*: Current status and future directions. Stud. Mycol..

[12] Dean R., Van Kan J.A., Pretorius Z.A., Hammond-Kosack K.E., Di Pietro A., Spanu P.D., Rudd J.J., Dickman M., Kahmann R., Ellis J. (2012). The top 10 fungal pathogens in molecular plant pathology. Mol. Plant Pathol..

[13] Hyde K.D., Cai L., Ehc M.K., Yang Y.L., Prihastuti H. (2009). *Colletotrichum*: A catalogue of confusion. Fungal Divers..

[14] Vieira W.A., Bezerra P.A., da Silva A.C., Veloso J.S., Câmara M.P.S., Doyle V.P. (2020). Optimal markers for the identification of *Colletotrichum* species. Mol. Phylogenet Evol..

[15] De Silva D.D., Crous P.W., Ades P.K., Hyde K.D., Taylor P.W.J. (2017). Life styles of *Colletotrichum* species and implications for plant biosecurity. Fungal Biol. Rev..

[16] Khakimov A., Salakhutdinov I., Omolikov A., Utaganov S. (2022). Traditional and current-prospective methods of agricultural plant diseases detection: A review. IOP Conf. Ser. Earth Environ. Sci..

[17] Kaur H., Singh R., Doyle V.P., Valverde R. (2021). A diagnostic TaqMan real-time PCR assay for in planta detection and quantification of *Colletotrichum theobromicola*, causal agent of boxwood dieback. Plant Dis..

[18] Du Y., Wang M., Zou L., Long M., Yang Y., Zhang Y., Liang X. (2021). Quantitative detection and monitoring of *Colletotrichum siamense* in rubber trees using real-time PCR. Plant Dis..

[19] Yang J., Duan K., Liu Y., Song L., Gao Q.H. (2022). Method to detect and quantify colonization of anthracnose causal agent *Colletotrichum gloeosporioides* species complex in strawberry by real-time PCR. J. Phytopathol..

[20] Wan M., Yang L., Zhang S., Gao J., Jiang L., Luo L. (2022). Real-time PCR for detection and quantification of *C. gloeosporioides* s.l. growth in *Stylosanthes* and *Arabidopsis*. Crop Prot..

[21] Chen Y.Y., Conner R.L., Gillard C.L., Mclaren D.L., Boland G.J., Balasubramanian P.M., Stasolla C., Zhou Q.X., Hwang S.F., Chang K.F. (2013). A quantitative real-time PCR assay for detection of *Colletotrichum lindemuthianum* in navy bean seeds. Plant Pathol..

[22] Tao G., Hyde K., Cai L. (2013). Species-specific real-time PCR detection of *Colletotrichum kahawae*. J. Appl. Microbiol..

[23] Sun T., Kong D., Teng S., Deng Z. (2017). Establishment of a TaqMan real-time PCR test method for detecting *Colletotrichum karstii*. Guizhou Agric. Sci. China.

[24] Wang C., Zhang X.J., Zhang X.L., Zhang W., Zhang Y., Li Y.W., Sun Y.F. (2016). Detection of *Colletotrichum orbiculare* by the real-time PCR. Plant Prot. China.

[25] He J., Sun M.L., Li D.W., Zhu L.H., Ye J.R., Huang L. (2023). A real-time PCR for detection of pathogens of anthracnose on Chinese fir using TaqMan probe targeting ApMat gene. Pest Manag. Sci..

[26] Dowling M., Peres N., Villani S., Schnabel G. (2020). Managing *Colletotrichum* on fruit crops: A “complex” challenge. Plant Dis..

[27] Hayden K.J., Rizzo D., Tse J., Garbelotto M., Matthiesen R.L., Schmidt C., Robertson A.E., Du Y., Wang M., Long M. (2004). Detection and quantification of *Phytophthora ramorum* from California forests using a real-time polymerase chain reaction assay. Phytopathology.

[28] Vieira W.A., Lima W.G., Nascimento E.S., Michereff S.J., Câmara M.P., Doyle V.P. (2017). The impact of phenotypic and molecular data on the inference of *Colletotrichum* diversity associated with Musa. Mycologia.

[29] Zou H., Li T., Zhang J., Shao H., Kageyama K., Feng W. (2024). Rapid detection of *Colletotrichum siamense* from infected tea plants using filter-disc DNA extraction and loop-mediated isothermal amplification. Plant Dis..

[30] Schena L., Nigro F., Ippolito A., Gallitelli D. (2004). Real-time quantitative PCR: A new technology to detect and study phytopathogenic and antagonistic fungi. Eur. J. Plant Pathol..

[31] Schoch C.L., Seifert K.A., Huhndorf S., Robert V., Spouge J.L., Levesque C.A., Chen W. (2012). Nuclear ribosomal internaltranscribed spacer (ITS) region as a universal DNA barcode marker for fungi. Proc. Natl. Acad. Sci. USA.

[32] Lücking R., Aime M.C., Robbertse B., Miller A.N., Ariyawansa H.A., Aoki T., Cardinali G., Crous P.W., Druzhinina I.S., Geiser D.M. (2020). Unambiguous identification of fungi: Where do we stand and how accurate and precise is fungal DNA barcoding?. IMA Fungus.

[33] Bhunjun C.S., Phukhamsakda C., Jayawardena R.S., Jeewon R., Promputtha I., Hyde K.D. (2021). Investigating species boundaries in *Colletotrichum*. Fungal Divers..

[34] Ginzinger D.G. (2002). Gene amplification using real-time quantitative PCR: An emerging technology hits the mainstream. Exp. Hematol..

