# Supplementary figures and images for: Development of a TaqMan Real-Time PCR for Early and Accurate Detection of Anthracnose Pathogen Colletotrichum siamense in Pachira glabra
Source: Plants (Basel). 2024 Apr 20;13(8):1149. doi: 10.3390/plants13081149 (PMC11054339; doi:10.3390/plants13081149)

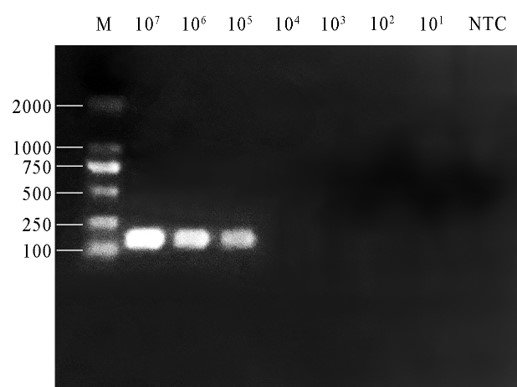

Supplement: Supplementary file 1 [file plants-13-01149-s001.zip › Figure S1.jpg]
